# Identifying the hub genes and immune infiltration related to pyroptosis in rheumatoid arthritis

**DOI:** 10.1097/MD.0000000000028321

**Published:** 2021-12-17

**Authors:** Wei Xie, Zhengyuan Wu

**Affiliations:** a Department of Orthopedics, Minzu Hospital of Guangxi Zhuang Autonomous Region, Nanning, China.; b Department of Hand Plastic Surgery, The First People's Hospital of Linping District, Hangzhou, China.

**Keywords:** bioinformatical analysis, Gene Expression Omnibus, immune infiltration, pyroptosis, rheumatoid arthritis

## Abstract

Rheumatoid arthritis (RA) is one of the most common autoimmune joint disorders globally, but its pathophysiological mechanisms have not been thoroughly investigated. Pyroptosis significantly correlates with programmed cell death. However, targeting pyroptosis has not been considered as a therapeutic strategy in RA due to a lack of systematic studies on validated biomarkers. The present study aimed to identify hub pyroptosis biomarkers and immune infiltration in RA. The gene expression profiles of synovial tissues were obtained from the Gene Expression Omnibus (GEO) database to identify differentially expressed pyroptosis genes (DEPGs). Meanwhile, the CIBERSORT algorithm was used to explore the association between immune infiltration and RA. Consequently, two hub DEPGs (*EGFR* and *JUN*) were identified as critical genes in RA. Through gene ontology and pathway enrichment analysis. *EGFR* and *JUN* were found to be primarily involved in the ErbB signaling pathway, PD-1 checkpoint pathway, GnRH signaling pathway, etc. Furthermore, for immune infiltration analysis, the pyroptosis genes *EGFR* and *JUN* were closely connected with four and one immune cell types, respectively. Overall, this study presents a novel method to identify hub DEPGs and their correlation with immune infiltration, which may provide novel perspectives into the diagnosis and treatment of patients with RA.

## Abstract 2

Supplemental Digital Content is available in the text

## 1 Introduction

Rheumatoid arthritis (RA) is a common arthritis disease worldwide and has been shown to severely impair the function of joints and affect the quality of life of the elderly.^[1]^ This disorder is a chronic multisystemic autoimmune disease mainly characterized by pannus formation and destructive synovitis.^[2]^ Distinct from slight leukocytic infiltration into the synovial membrane of osteoarthritis,^[3]^ various lymphocytes and inflammatory cells, including synovial fibroblasts, endothelial cells, T/B cells, osteoclasts, monocytes/macrophages, and dendritic cells, infiltrate the RA synovial membrane.^[4]^ These inflammatory cells form a complex cellular network, both by the release of inflammatory cytokines and cell–cell interactions,^[5]^ which eventually results in the destruction of cartilage and progression of RA. In recent years, studies have provided a deeper understanding of the pathophysiology of RA. However, only a few screening biomarkers and therapeutic interventions of significance in clinical treatment are available. Therefore, the elucidation of more unique biomarkers for RA is urgently needed for the accurate identification of patients and development of therapies

Pyroptosis is a new type of lytic cell death, which is characterized by bubble-like protrusions and cellular swelling.^[6]^ The gasdermin family is regarded as the main executor of pyroptosis.^[7]^ During the process of pyroptosis, cells can form various types of vesicles, after which gasdermin is multimerized and sheared, forming a number of 10 to 20 nm pores in the cell membrane. With cell contents continuously flowing out from the membrane pores, cells produce apoptotic vesicle-like protrusions and gradually swell until they rupture.^[8,9]^ In recent years, studies have shown that the process of pyroptosis is also significantly associated with inflammatory cytokines released through the caspase-1-dependent pathway, subsequently causing the pathogenesis of RA.^[10]^ Unlike cell apoptosis, pyroptosis cannot only propagate and activate inflammasomes to trigger a strong inflammatory response but also lead to immune system activation, especially the activation of T helper 1 (Th1) and T helper 2 (Th2) cells,^[10,11]^ which is the main pathology feature of RA.^[12]^ Thus, it is accepted that pyroptosis is associated with RA progression; however, its specific role in RA is still poorly understood.

With the development of microarray technologies, bioinformatics analyses have been widely employed to identify disease-specific biomarkers, explore the molecular mechanisms, and reveal significant epigenetic and genetic alterations in RA.^[13]^ The weighted gene co-expression network (WGCN) is a frequently used method, focusing on gene sets rather than individual gene expression, to understand the gene association patterns between different phenotypic traits.^[14,15]^ During WGCN analysis (WGCNA), gene expression data can be used to construct a powerful scale-free network to identify hub genes for mechanism evaluation and clinical diagnosis. Differential gene expression analysis of transcriptional data is another powerful tool that provides quantitative expression-level changes between two subgroups.^[16]^

Up to date, most hub gene identification methods for RA have mainly been constructed based on a simplified differential analysis, and no method has systemically explored pyroptosis genes in RA or their connections with immune cells. In this study, candidate pyroptosis genes that were differentially expressed in RA versus a normal control were determined using WGCNA and differential gene expression analysis to enhance the discriminatory ability of highly connected genes. Subsequently, the CIBERSORT algorithm method was also used to characterize hub genes significantly related to immune infiltration in RA synovium tissues. The work presented here aimed to uncover a novel method to identify hub pyroptosis-associated genes and their correlation with immune infiltration, which may provide novel insight into the diagnosis and therapeutic targets of RA.

## 2 Materials and methods

### 2.1 Raw data acquisition and preprocessing

Gene expression profiles of synovial tissues from joints were acquired from the Gene Expression Omnibus database (GEO; ID: GSE12021, GSE55235, GSE55457, and GSE55584; https://www.ncbi.nlm.nih.gov/geo/). In the GSE12021 data set, the same specimen was analyzed by two different detection platforms (GPL96 and GPL97) simultaneously. In view of other cohorts using the GPL96 method, we excluded the data of GPL97 from this research analysis; this was not expected to have any impact on our analysis. After samples were separated with different detection platforms, the remaining specimens, including 29 healthy control samples and 45 RA samples, were all merged as one profile to explore hub genes. All samples restricted to joint synovial tissues were collected from patients with RA after synovectomy surgery or from macroscopically normal knee joints during early postmortem examination at the Department of Orthopedics, Jena University Hospital; Department of Orthopedics/Institute of Pathology, University of Leipzig; and Department of Orthopedics/Institute of Pathology/Department of Rheumatology and Clinical Immunology, Charité-Universitätsmedizin. The clinical characteristics of tissue samples are presented in Table S1, Supplemental Digital Content, http://links.lww.com/MD2/A773. Approval by the Ethics Committee was not necessary because all data were collected from publicly available databases (GEO). As previously described, log2-transformation and normalization by the “sva” package in R were performed to remove batch effects.^[13,17]^ Furthermore, 146 protein domains for the specific pyroptosis genes were collected from the GeneCards database (https://www.genecards.org).

### 2.2 Differential expression analysis and interaction with the modules of interest

Candidate differential expressed genes (DEGs) were identified using differential expression analysis using the “limma” package. Genes with a false discovery rate (FDR) < 0.05 and |log2 fold change (FC)| > 1 were selected as candidate DEGs based on previous methods^[18]^ and were visualized by volcano plots and heatmaps using the “ggplot2” and “pheatmap” packages, respectively.^[19]^

### 2.3 Gene ontology (GO) and Kyoto encyclopedia of genes and genomes (KEGG) enrichment analyses

The Database for Annotation, Visualization and Integrated Discovery version 6.8^[20]^ was employed to determine the biological functions of hub genes. The biological process (BP), molecular function (MF), and cellular component (CC) were applied in GO enrichment analyses. Statistical significance was considered when both FDR and *P*-values were <.05.

### 2.4 Construction of the WGCN and hub module identification

The complete gene expression profiles were utilized to construct a gene co-expression network in RA by the “WGCNA” package.^[21,22]^ All genes were subjected to pairwise Pearson's correlation coefficients, and a weighted adjacency matrix was identified with the following formula: amn=|cmn|β (cmn = Pearson's correlation between gene n and gene m; amn = adjacency between genes m and n). Then, a suitable soft-threshold parameter “β” was determined to emphasize strong gene correlations and penalize weak correlations. A topological overlap matrix (TOM) transformed adjacencies. Using TOM-based dissimilarity measures, the average linkage hierarchical clustering was used to construct the gene dendrogram with at least a 50-module size. Meanwhile, dissimilarities of module eigengenes were calculated. Consequently, the light green module was identified to be most relevant to RA clinical traits, and genes within the light green module were labeled as candidates. The overlapping genes between WGCN, candidate DEGs, and pyroptosis genes list were considered the “real” pyroptosis-associated DEGs (DEPGs), which were visualized in a Venn diagram using the “VennDiagram” package.^[23]^

### 2.5 Immune infiltration analysis by CIBERSORT

The CIBERSORT algorithm (https://cibersort.stanford.edu/) is a widely applied method for calculating the proportions of 22 types of leukocytes in complex tissues.^[24]^ In the present study, CIBERSORT analysis was performed to characterize immune cell infiltration in RA synovial tissues and screened with a *P*-value < .05. The composition of immune cells in each sample was visualized by a barplot and heatmap, and the infiltration levels of each cell between RA and healthy controls were visualized by a vioplot.

## 3 Results

### 3.1 Candidate DEG identification

A total of 300 genes selected from the differential expression analysis, comprising 161 downregulated and 139 upregulated genes, were considered candidate DEGs (Figure S1A and B, Supplemental Digital Content, http://links.lww.com/MD2/A774).

### 3.2 Functional enrichment analysis of candidate DEGs

GO analysis showed that, in terms of BP, the candidate DEGs were significantly enriched in regulation of lymphocyte activation, leukocyte activation and migration, and antigen receptor-mediated signaling pathway (Table 1). With respect to MF, genes were mainly enriched in receptor ligand activity, chemokine activity, and cytokine activity, whereas in terms of CC, genes were enriched in external side of plasma membrane, clathrin-coated vesicle, and MHC class II protein complex. KEGG pathway analysis showed that candidate DEGs were enriched in rheumatoid arthritis, primary immunodeficiency, Th17 cell differentiation, and TNF signaling pathway (Table 1).

**Table 1:** GO and KEGG enrichment analysis of DEGs (top 10 terms of each category were listed).

Platform	ID	Description	Adj. *P*	Count
GO: BP	0051249	Regulation of lymphocyte activation	4.99E–10	32
GO: BP	0002696	Positive regulation of leukocyte activation	6.49E–10	28
GO: BP	0051251	Positive regulation of lymphocyte activation	7.85E–10	26
GO: BP	0050867	Positive regulation of cell activation	7.85E–10	28
GO: BP	0050851	Antigen receptor-mediated signaling pathway	1.07E–09	25
GO: BP	0007159	Leukocyte cell–cell adhesion	3.70E–09	25
GO: BP	0050900	Leukocyte migration	4.63E–09	30
GO: BP	0030098	Lymphocyte differentiation	7.62E–09	25
GO: BP	0042110	T cell activation	1.62E–08	28
GO: BP	0042113	B cell activation	1.62E–08	23
GO: CC	0009897	External side of plasma membrane		
	5.71E–06	22		
GO: CC	0030665	Clathrin-coated vesicle membrane	0.00053741	10
GO: CC	0030136	Clathrin-coated vesicle	0.00399194	11
GO: CC	0042613	MHC class II protein complex	0.00399194	4
GO: CC	0030662	Coated vesicle membrane	0.01126779	10
GO: CC	0030669	Clathrin-coated endocytic vesicle membrane	0.01442042	5
GO: CC	0042611	MHC protein complex	0.01442042	4
GO: CC	0042571	Immunoglobulin complex, circulating	0.01609076	6
GO: CC	0001772	Immunological synapse	0.04653418	4
GO: CC	0045334	Clathrin-coated endocytic vesicle	0.04902248	5
GO: MF	0048018	Receptor ligand activity	8.98E–05	23
GO: MF	0008009	Chemokine activity	8.98E–05	
	8			
GO: MF	0005125	Cytokine activity	8.98E–05	15
GO: MF	0003823	Antigen binding	0.00035107	12
GO: MF	0042379	Chemokine receptor binding	0.00035107	8
GO: MF	0001228	DNA-binding transcription activator activity, RNA polymerase II-specific	0.00035107	20
GO: MF	0005126	Cytokine receptor binding	0.0009742	15
GO: MF	0001664	G protein-coupled receptor binding	0.00280182	14
GO: MF	0048020	CCR chemokine receptor binding	0.01869154	5
GO: MF	0001968	Fibronectin binding	0.02665551	4
KEGG	hsa05323	Rheumatoid arthritis	1.56E–06	13
KEGG	hsa05340	Primary immunodeficiency	2.47E–05	8
KEGG	hsa04659	Th17 cell differentiation	2.47E–05	12
KEGG	hsa04668	TNF signaling pathway	3.06E–05	12
KEGG	hsa04060	Cytokine-cytokine receptor interaction	3.62E−05	19
KEGG	hsa05166	Human T-cell leukemia virus 1 infection	4.88E−05	16
KEGG	hsa04064	NF-kappa B signaling pathway	6.07E−05	11
KEGG	hsa04380	Osteoclast differentiation	6.47E−05	12
KEGG	hsa04658	Th1 and Th2 cell differentiation	0.00011066	10
KEGG	hsa04640	Hematopoietic cell lineage	0.0001939	10

### 3.3 Identification of hub modules by constructing a WGCN

As shown in Figure S2A, Supplemental Digital Content, http://links.lww.com/MD2/A775 synovial tissues from healthy joints and RA samples were included in WGCNA. To construct a scale-free network, the soft threshold was identified by β = 6 (scale-free *R*^2^ = 0.85) (Figure S2B, Supplemental Digital Content, http://links.lww.com/MD2/A775), and a total of 17 co-expressed modules were identified (Figure S2C, Supplemental Digital Content, http://links.lww.com/MD2/A775). Among all modules, the light green module had the closest connection with RA formation (Figure S2D, Supplemental Digital Content, http://links.lww.com/MD2/A775) and was identified as key module of interest for further explorations with 70 different genes.

### 3.4 Identification of overlapping DEPGs and correlation with immune infiltration

**Figure 1:**
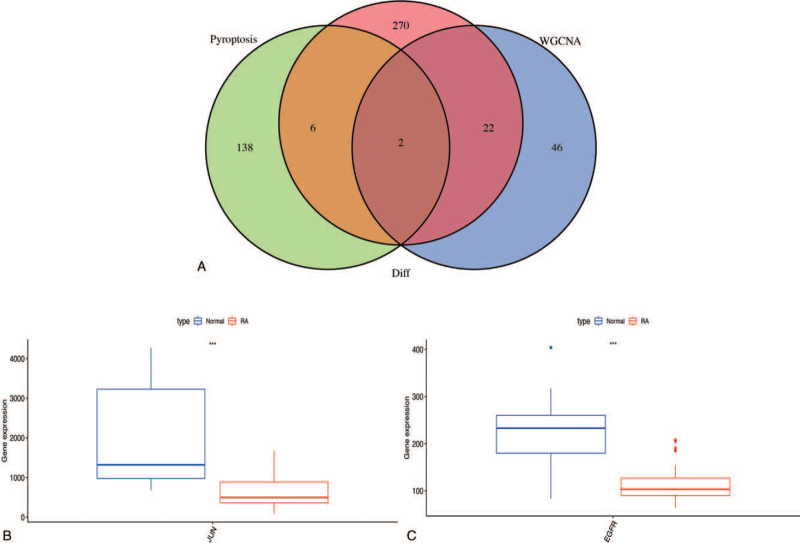
Identification of hub DEPGs. (A) The Venn diagram of genes among pyroptosis gene list, candidate DEGs, and WGCNA of light green module. The box plot shows mRNA expression pattern of *JUN* (B) and *EGFR* (C) in normal tissues and RA synovial samples.

The number distribution of the co-expressed genes from WGCNA, candidate DEGs, and pyroptosis-associated genes is displayed in Figure 1A. Two overlapping genes (epidermal growth factor receptor gene [*EGFR*] and Jun proto-oncogene, AP-1 transcription factor subunit gene [*JUN*]) were extracted for further exploration. The gene expression level comparison of hub genes indicated that both *EGFR* and *JUN* had significantly low expression in RA tissues (Fig. 1B and C).

**Figure 2:**
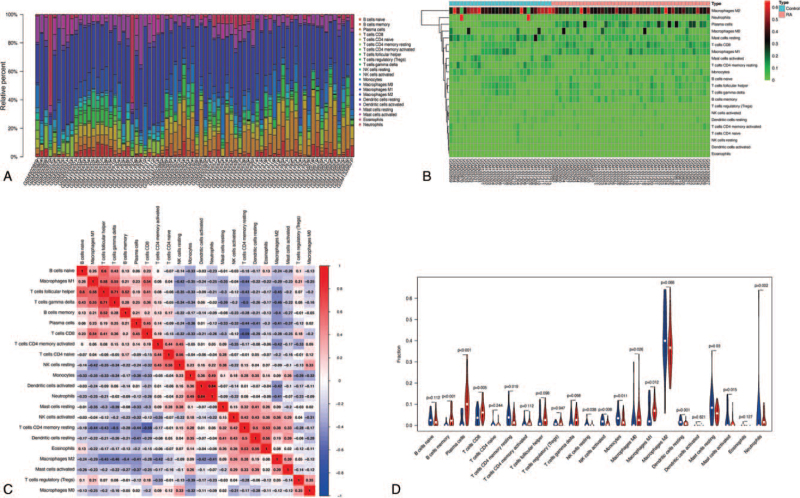
Immune infiltration analysis. Barplot (A) and heatmap (B) showed the composition of 22 subpopulations of immune cells in RA synovial and normal controls. (C) Heatmap showed the correlation between 22 kinds of immune cells and numeric in each tiny box indicating the *P* value of correlation between two types of cells. (D) The violin plot showed the difference of immune infiltration between RA (red color) and normal controls (blue color).

**Figure 3:**
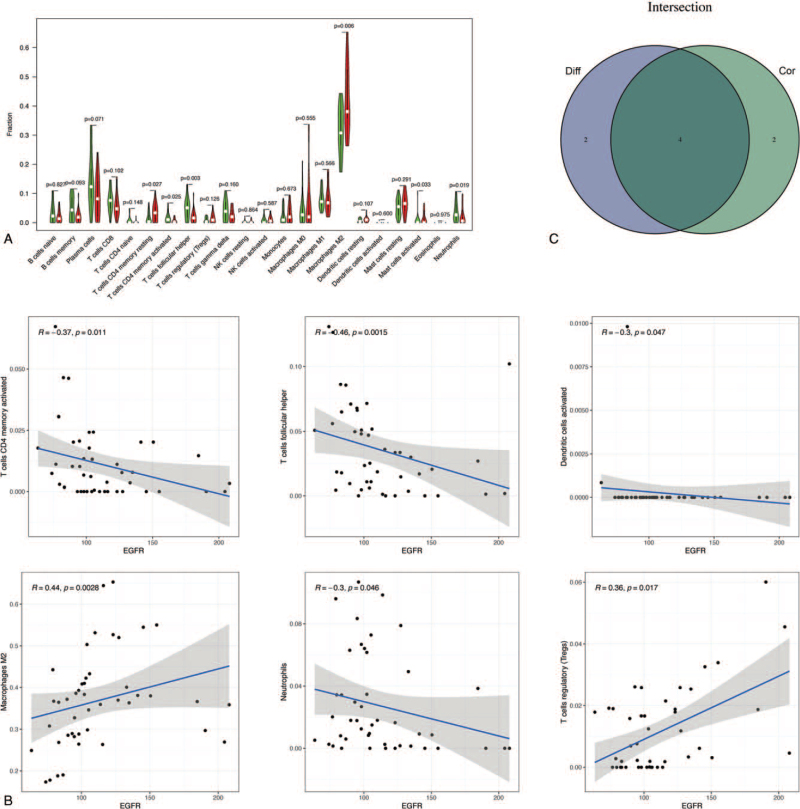
Correlation of immune infiltration with *EGFR* expression in RA. (A) Violin plot showed the ratio differentiation of 22 kinds of immune cells between RA samples with low (green color) or high (red color) *EGFR* expression. (B) Scatter plot showed the correlation of 6 kinds of immune cells proportion with the *EGFR* expression (*P* < .05). The blue line in each plot was fitted linear model indicating the proportion tropism of the immune cell along with *EGFR* expression. (C) Venn plot of immune cells codetermined by the difference and correlation analysis.

**Figure 4:**
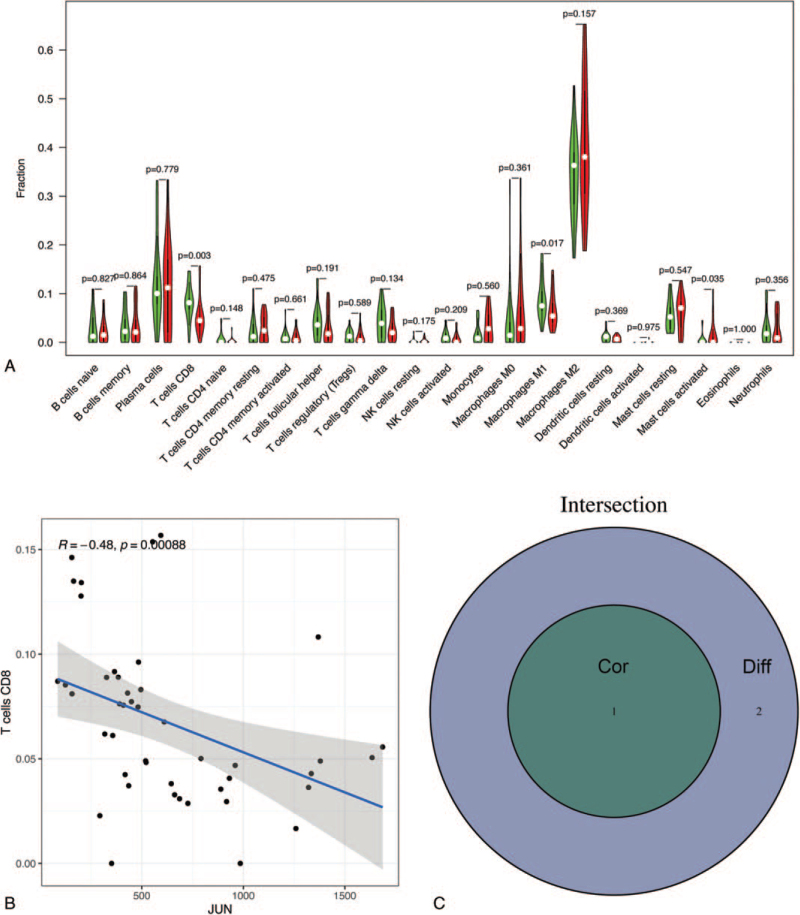
Correlation of immune infiltration with JUN expression in RA. (A) Violin plot showed the ratio differentiation of 22 kinds of immune cells between RA samples with low (green color) or high (red color) *JUN* expression. (B) Scatter plot showed the correlation of CD8 T cells proportion with the *JUN* expression. The blue line in each plot was fitted linear model indicating the proportion tropism of the immune cell along with *JUN* expression. (C) Venn plot of immune cells codetermined by the difference and correlation analysis.

To further confirm the interactions between DEPG expression and immune infiltration, the CIBERSORT algorithm was first applied to analyze the proportion of 22 immune cells in RA. As shown in Figure 2, memory B cells, plasma cells, CD8 T cells, M0 macrophages, M1 macrophages, activated mast cells, and neutrophils were abundant in RA synovial membrane compared to those in healthy controls. In contrast, the proportions of resting memory CD4 T cells, resting NK cells, activated NK cells, monocytes, resting dendritic cells, and resting mast cells were relatively lower in RA tissues. Meanwhile, the difference and correlation analysis also revealed that the infiltrating levels of four and one kinds of immune cells were significantly connected with *EGFR* and *JUN* expression levels, respectively (*P* < .05; Figs. 3 and 4). Among them, M2 macrophages were positively correlated with EGFR expression, whereas activated CD4 memory T cells, follicular helper T cells, and neutrophils were negatively correlated with *EGFR* expression. Although activated dendritic cells and regulatory T cells (Tregs) were also associated with *EGFR* expression, their expression in RA was not significantly different from that in normal tissues. With respect to the expression of JUN, only CD8 T cells were significantly associated with it. These results indicated that EGFR and JUN might participate in the progression of RA through the modulation of immune cell infiltration.

### 3.5 GO and KEGG enrichment analysis of EGFR and JUN

**Figure 5:**
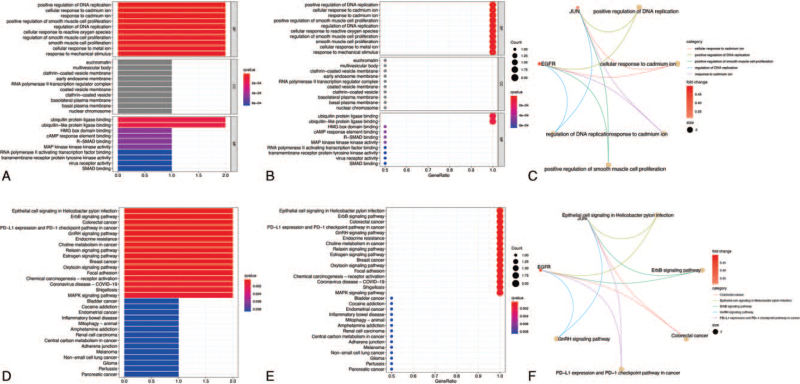
Functional enrichment analysis. (A–C) GO enrichment terms of hub DEPGs in biological process (BP), cellular component (CC), and molecular function (MF). (D–F) KEGG enrichment terms of hub DEPGs. In each bubble plot, the size of the dot represents the number of enriched genes.

As shown in Figure 5A–C, the two hub DEPGs are involved in some critical BPs, such as regulation of DNA replication, response to cadmium ions, and smooth muscle cell proliferation. DEPGs were also found to be enriched in euchromatin, multivesicular body, and clathrin-coated vesicle members in the CC category and in ubiquitin protein ligase binding, HMG box domain binding, and cAMP response element binding in the MF category. Moreover, the results of KEGG enrichment analysis indicated that DEPGs played a possible role in numerous tumors, such as colorectal cancer, breast cancer, and bladder cancer (Fig. 5D–F). Furthermore, these genes were also significantly connected with pathways of the ErbB signaling pathway, PD-1 checkpoint pathway, and GnRH signaling pathway.

## 4 Discussion

As the most common osteoarthropathy, RA results in irreversible bone erosion and cartilage destruction. Without timely and effective treatment, RA tends to severely affect the function of patients’ joints.^[25]^ In recent years, increasing studies have focused on the modulatory effects of synovitis in RA. Synovial inflammation leads to the formation of inflammatory pannus, resulting in cartilage erosion and a recalcitrance to therapies.^[26,27]^ The evaluation of the potential mechanism of synovitis in RA may help improve diagnosis and treatment. The development of high-throughput technologies has enabled scientists to discover novel therapeutic targets and to acquire a deeper understanding of the molecular mechanisms of RA^[28]^; however, the distinctive pathogenesis and detailed mechanism of RA progression in the synovium remains elusive.

To fill the present knowledge gap, the gene profiles of 74 synovial tissues were collected; a co-expression network in RA was constructed; and differential expression analysis was applied to identify the DEPGs. As a result, two hub pyroptosis-associated genes, that is, *EGFR* and *JUN*, were identified as differentially expressed biomarkers. Although *EGFR* and *JUN* have been described to be connected with RA development, no studies thus far have systemically determined hub pyroptosis genes in RA. Therefore, our analysis might generate a novel perspective into the relationship between pyroptosis and RA and identify valuable pyroptosis-linked biomarkers for personalized treatment.

As a vital mediator of synovial hyperplasia, *EGFR*, also known as ErbB/HER, plays a key role in angiogenesis regulation of several tissues’ hyperplastic growth, as well as RA pathology.^[29]^ The specific role of EGFR in contributing to RA progression is ascribed to its promotion of endothelial cell proliferation and cytokine production of synovial fibroblasts and inhibition of osteoclast formation.^[30,31]^ The present results are in line with studies that have demonstrated that EGFR is differentially expressed in synovial lining, sub-synovial fibroblasts, and vascular endothelial cells of RA.^[29,32]^ The inhibition of *EGFR* by cetuximab^[33]^ and erlotinib^[29]^ can effectively ameliorate the symptoms of RA, which suggests that targeting *EGFR* might be promising in the treatment of patients with RA. The activation of *EGFR* is initiated by binding one of at least six ligands, including amphiregulin, transforming growth factor alpha, EGF, betacellulin, epiregulin, and heparin-binding EGF-like growth factor, to the extracellular domain.^[30]^ Recent studies have indicated that *EGFR* ligands are also differentially expressed in the serum and synovium of RA patients.^[34,35]^ Interestingly, the EGFR ligands amphiregulin and EGF can promote angiogenesis through VEGF, inflammation through interleukin (IL)-6, cartilage destruction through matrix metallopeptidase 3 (MMP-3), and neutrophil and monocyte migration through IL-8 and monocyte chemotactic protein-1 (MCP-1).^[36]^ Thus, all these results indicate that *EGFR* can serve as a promising novel target for therapeutic intervention in patients with RA.

The transcription factor family activator protein 1 (AP-1) is involved in various cellular processes, such as apoptosis, proliferation, and differentiation.^[37]^ As a member of AP-1, *JUN* also participates in the modulation of the RA inflammatory process by synergistically acting with NF-κB, which can result in the activation of proinflammatory cytokines or chemokines.^[37,38]^ Indeed, *JUN* also mediates a wide series of macrophage activation signals in RA, including inflammatory stimuli and Th1 and Th2 cytokines.^[39]^ Next, the proinflammatory mediator cytochrome c oxidase subunit-2 (COX-2), which is directly controlled by *JUN* in macrophages,^[40]^ elevates the production of PGE_2_ and ultimately causes cartilage degeneration and bone resorption.^[41,42]^ Another transcriptional target of *JUN* in macrophages is the Arg1 pathway, which contributes to the resolution of inflammation and tissue repair in arthritis.^[43]^ Hence, *JUN* might counteract the resolution of inflammation possibly by downregulating the expression of Arg1. Overall, we can conclude that both *EGFR* and *JUN* influence the severity of RA, act as hub pyroptosis-associated targets in the pathological progression of RA, and deserve more research attention.

The identified DEPGs were also assessed using functional enrichment analysis. Most genes were found to be closely related to various pathways, such as the ErbB signaling pathway, PD-1 checkpoint pathway, and GnRH signaling pathway, all of which are involved in the progression of RA.^[44–46]^ In addition, considering the vital role of immune cell infiltration in RA, the CIBERSORT algorithm method was further applied. Several immune cells were significantly different between the RA synovium and control. Among immune cells, M2 macrophages, activated CD4 memory T cells, follicular helper T cells, and neutrophils were significantly correlated with *EGFR* expression levels; meanwhile, CD8 T cells were significantly associated with *JUN* expression levels, which suggested that *EGFR and JUN* might modulate RA synovial hyperplasia and progression by acting on these types of immune cells. However, the exact relationship between *EGFR*, *JUN*, and immune cells and the concrete effects of hub pyroptosis genes on synovial immune infiltration need to be confirmed by further studies.

This study had a few limitations. First, this is a retrospective analysis; thus, prospective approaches to confirm the results are warranted. Second, there was a lack of experimental explorations to confirm the bioinformatics results. Future experiments should aim to derive mechanistic insights into the identified pyroptosis genes and their connections with RA development.

To summarize, two hub pyroptosis-associated genes (*EGFR* and *JUN*) that possibly play a critical role in RA pathogenesis were identified, and functional enrichment analysis of the identified biomarkers provided a potential mechanism for clarifying RA development. The findings also showed that *EGFR* and *JUN* might underlie the modulation of immune cells in RA synovial tissues. Further investigations should verify the accuracy of a combined analysis of *EGFR* and *JUN* expression levels and immune infiltration profiles in patients with RA (Supplementary File 2, http://links.lww.com/MD2/A776).

## Author contributions

**Conceptualization:** Zhengyuan Wu.

**Data curation:** Wei Xie.

**Formal analysis:** Wei Xie.

**Methodology:** Wei Xie.

**Writing – original draft:** Wei Xie, Zhengyuan Wu.

**Writing – review & editing:** Zhengyuan Wu.
